# Comparative Efficacy of Fluoroscopically Guided vs. Landmark-Based Injections in the Management of Ischial Bursitis: A Pilot Study

**DOI:** 10.7759/cureus.77185

**Published:** 2025-01-09

**Authors:** Jamal Hasoon, Giustino Varrassi, Omar Viswanath

**Affiliations:** 1 Department of Anesthesia, Critical Care and Pain Medicine, UTHealth, McGovern Medical School, Houston, USA; 2 Pain Medicine, Fondazione Paolo Procacci, Rome, ITA; 3 Pain Management, Mountain View Headache and Spine Institute, Phoenix, USA

**Keywords:** chronic pain, fluoroscopy intervention, interventional pain, ischial bursa injection, ischial bursitis

## Abstract

Introduction

Ischial bursitis is a challenging chronic pain condition often resistant to conservative treatments. Ischial bursa injections, including fluoroscopically guided and landmark-based approaches, are commonly used when first-line interventions fail. This study aims to compare the efficacy of these two techniques in providing pain relief and improving function in patients with refractory ischial bursitis.

Methods

A retrospective analysis was conducted using electronic medical records of nine patients with refractory ischial bursitis treated between April 1, 2023, and November 30, 2024. Patients were categorized based on the injection technique: fluoroscopically guided (n=4) or landmark-based (n=5). Pain relief was assessed at follow-up appointments using patient-reported outcomes. Statistical analyses were performed to compare the mean pain relief between groups.

Results

The fluoroscopically guided group demonstrated significantly greater pain relief, with a mean improvement of 86.25% ± 11.09%, compared to 55.00% ± 13.23% in the landmark-based group (p < 0.05). Patients receiving fluoroscopic injections consistently reported ≥75% relief, while the landmark-based group experienced more variable outcomes, ranging from 40% to 75%. These findings highlight the superior efficacy and consistency of image-guided injections.

Conclusion

Fluoroscopically guided ischial bursa injections provide greater pain relief compared to landmark-based injections in patients with refractory ischial bursitis. The precise delivery of medication under image guidance appears to enhance therapeutic outcomes. Future studies with larger sample sizes and randomized designs are warranted to validate these findings and refine treatment protocols for this patient population.

## Introduction

Ischial bursitis is an inflammatory condition involving the ischial bursa, located between the ischial tuberosity and the adjacent soft tissues of the gluteal region [1,2]. It commonly presents as localized pain in the lower buttocks, sometimes radiating down the posterior thigh, and is exacerbated by prolonged sitting or direct pressure [3,4]. Although relatively less common than other forms of bursitis, ischial bursitis can cause significant discomfort and limitations in daily activities, particularly in the seated position. The condition is often associated with repetitive trauma, prolonged sitting on hard surfaces, or certain athletic activities that place pressure on the ischial tuberosities [2-4].

Management of ischial bursitis typically begins with conservative approaches, including physical therapy (PT), oral analgesics, nonsteroidal anti-inflammatory drugs (NSAIDs), muscle relaxants, and activity modification. While these therapies can be effective, a subset of patients continues to experience refractory pain despite adherence to standard treatment modalities. For these patients, targeted ischial bursa injections may provide relief by delivering medication directly to the site of inflammation. Injections for ischial bursitis can be performed using either a landmark-based approach or under image guidance, depending on the clinical scenario and provider preference.

This pilot study describes nine patients with persistent ischial bursitis who are unresponsive to conservative therapies. Four patients underwent fluoroscopically guided ischial bursa injections while five patients underwent landmark-based injections.

## Materials and methods

This study utilized a retrospective chart review to evaluate the efficacy of ischial bursa injections in patients with refractory ischial bursitis. Data were retrieved from electronic medical records of patients treated between April 1, 2023, and November 30, 2024, across multiple clinical sites. Patients included in the study were those with a documented diagnosis of ischial bursitis who had failed conservative treatments and subsequently underwent ischial bursa injections.

All landmark-based ischial bursa injections were performed by the same physician experienced in landmark-based techniques. The patient was positioned in either a prone or lateral decubitus position for the procedure. The ischial tuberosity was palpated to identify the painful area corresponding to the bursa. After the skin was thoroughly cleansed with an antiseptic solution, a 25-gauge needle was inserted perpendicularly toward the bursa. Aspiration confirmed the absence of vascular entry before injecting a mixture of local anesthetic and corticosteroid.

All fluoroscopy-guided ischial bursa injections were performed by two interventional pain physicians experienced in this technique. The patient was positioned prone for the procedure. Under fluoroscopic guidance, the ischial tuberosity was identified, and the target area corresponding to the bursa was localized. After the skin was thoroughly cleansed with an antiseptic solution, a 22-gauge needle was advanced with fluoroscopic imaging to ensure accurate placement near the bursa. Aspiration confirmed the absence of vascular entry, followed by the injection of a mixture of local anesthetic and corticosteroid.

The collected data were categorized based on the injection technique utilized: fluoroscopically guided or landmark-based. Each patient’s clinical history was reviewed to identify prior treatment modalities, including PT, oral analgesics, and any additional interventional pain management procedures. This allowed for an understanding of the treatment pathways leading up to the injections. Patient outcomes were assessed through follow-up records, which documented subjective reports of pain relief and functional improvement. Follow-up intervals ranged from 4 to 12 weeks, depending on the availability of clinical documentation. Pain relief was recorded as a percentage improvement from baseline, while functional outcomes included mobility and the ability to perform daily activities.

To ensure data accuracy, inclusion criteria required clear documentation of the injection technique and diagnosis. Records with incomplete or ambiguous information were excluded. All collected data were de-identified and anonymized to uphold patient confidentiality and ensure compliance with ethical research standards. Descriptive statistics, including mean and standard deviation, were used to summarize pain relief outcomes, while a two-sample t-test compared the mean pain relief between fluoroscopically guided and landmark-based groups.

## Results

Nine patients with refractory ischial bursitis were treated with ischial bursa injections after failing conservative therapies. Four patients underwent fluoroscopically guided injections, each receiving 3 mL of 0.25% bupivacaine combined with 40 mg of triamcinolone per bursa. For patients with bilateral bursitis, this solution was administered to each affected bursa for a total of 6 mL of 0.25% bupivacaine combined with 80 mg of triamcinolone divided among the bursas. Five patients received landmark-based injections with the same solution.

Fluoroscopically guided injections

Patient 1

This patient had bilateral ischial bursitis, reporting persistent pain despite PT, acetaminophen, NSAIDs, and cyclobenzaprine. Previous sacroiliac joint injections were ineffective, and the patient relied on support pillows for sitting. Bilateral fluoroscopically guided injections were administered. At a 6-week follow-up, the patient reported complete (100%) pain relief and significant improvements in sitting tolerance and mobility.

Patient 2

This patient with right-sided ischial bursitis failed trials of PT, acetaminophen, NSAIDs, gabapentin, and acetaminophen with codeine. A right-sided fluoroscopic injection provided 75% pain relief at an 8-week follow-up, which allowed for the discontinuation of opioid medications and satisfactory pain control.

Patient 3

This patient, with bilateral ischial bursitis, failed multiple conservative therapies, including PT, acetaminophen, cyclobenzaprine, tizanidine, and tramadol. Following bilateral fluoroscopic injections, the patient reported 90% relief at a 12-week follow-up, with notable functional improvements in daily activities and sitting tolerance.

Patient 4

A patient with right-sided ischial bursitis had unrelieved pain despite PT, acetaminophen, cyclobenzaprine, and etodolac. A fluoroscopically guided injection provided 80% pain relief at a 9-week follow-up, alongside substantial functional improvements in daily activities and mobility.

Landmark-based injections

Patient 1

This patient presented with left-sided ischial bursitis and previously failed conservative treatments, including PT, acetaminophen, ibuprofen, celecoxib, baclofen, and hydrocodone. A sacroiliac joint injection had also been ineffective. Following a left-sided landmark-based injection, the patient reported 50% relief at a 4-week follow-up but required a repeat fluoroscopic injection at 10 weeks. The patient did not present for a follow-up appointment after a repeat injection.

Patient 2

A patient with bilateral ischial bursitis failed trials of PT, acetaminophen, ibuprofen, and tizanidine. Bilateral landmark-based injections provided 75% relief at an 8-week follow-up, along with significant improvements in mobility and daily function.

Patient 3

This patient had bilateral ischial bursitis and previously failed conservative treatments, including PT, acetaminophen, ibuprofen, meloxicam, etodolac, and tramadol. Landmark-based injections resulted in 60% pain relief at an 8-week follow-up, with functional improvements in daily activities.

Patient 4

A patient with right-sided ischial bursitis failed conservative measures, including PT, acetaminophen, celecoxib, and meloxicam. Previous epidural steroid and trochanteric bursa injections provided only transient relief. Following a landmark-based injection, the patient reported 50% pain relief at a 10-week follow-up, with improved daily function.

Patient 5

This patient, with right-sided ischial bursitis, failed treatments including PT, acetaminophen, meloxicam, cyclobenzaprine, and tramadol. A landmark-based injection provided 40% relief at a 10-week follow-up.

The results of this study are summarized below (Table 1).

**Table 1:** Ischial Bursitis Patients Injection Outcomes The table identifies each patient and the injection method used. (F) indicates fluoroscopic-guided injections, and (L) indicates landmark-based injections. NSAIDS: nonsteroidal anti-inflammatory drugs

Patient	Category	Medications Tried	Injection Outcome
Patient 1 (F)	Fluoroscopic	Physical therapy, acetaminophen, NSAIDs, cyclobenzaprine	100% relief at 6 weeks
Patient 2 (F)	Fluoroscopic	Physical therapy, acetaminophen, NSAIDs, gabapentin, Tylenol with codeine	75% relief at 8 weeks
Patient 3 (F)	Fluoroscopic	Physical therapy, acetaminophen, cyclobenzaprine, tizanidine, tramadol	90% relief at 12 weeks
Patient 4 (F)	Fluoroscopic	Physical therapy, acetaminophen, cyclobenzaprine, etodolac	80% relief at 9 weeks
Patient 1 (L)	Landmark	Physical therapy, acetaminophen, ibuprofen, celecoxib, baclofen, hydrocodone	50% relief at 4 weeks; required repeat injection at 10 weeks
Patient 2 (L)	Landmark	Physical therapy, acetaminophen, ibuprofen, tizanidine	75% relief at 8 weeks
Patient 3 (L)	Landmark	Physical therapy, acetaminophen, ibuprofen, meloxicam, etodolac, tramadol	60% relief at 8 weeks
Patient 4 (L)	Landmark	Physical therapy, acetaminophen, celecoxib, meloxicam	50% relief at 10 weeks
Patient 5 (L)	Landmark	Physical therapy, acetaminophen, meloxicam, cyclobenzaprine, tramadol	40% relief at 10 weeks

**Figure 1:**
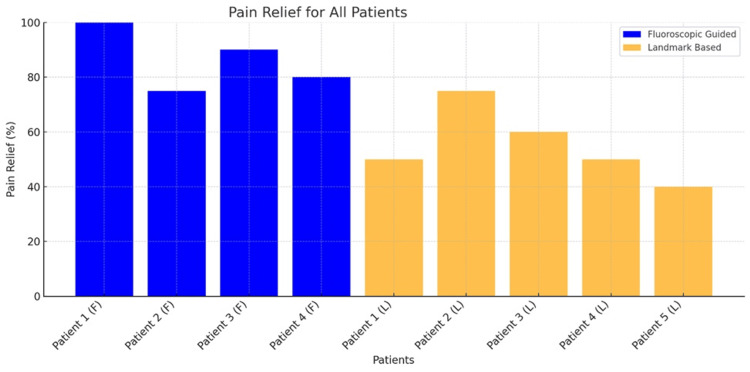
Pain Relief for All Patients This figure illustrates the pain relief achieved by patients undergoing fluoroscopic-guided and landmark-based ischial bursa injections for refractory ischial bursitis. Patients in blue represent those who received fluoroscopically guided injections, while patients in orange represent those who underwent landmark-based injections.

The average pain relief in the fluoroscopically guided group was 86.25% ± 11.09%, significantly higher than the 55.00% ± 13.23% observed in the landmark-based group (p < 0.05). Fluoroscopic injections demonstrated consistent outcomes, with all patients reporting ≥75% relief. In contrast, landmark-based injections showed variable efficacy, with relief ranging from 40% to 75%. This small-scale study highlights the potential advantages of fluoroscopic-guided ischial bursa injections over landmark-based approaches in the management of refractory ischial bursitis. These results suggest that precise delivery of medication under imaging may play a vital role in achieving optimal outcomes. The results of these injections are displayed visually below (Figure 1).

## Discussion

Numerous studies have evaluated the benefits of image guidance for joint injections. Several studies have demonstrated minimal benefits of image guidance for smaller joints, including shoulder injections [5]. However, additional studies have demonstrated improved outcomes and accuracy with the use of image guidance in musculoskeletal injections, especially when used for deeper structures such as hip injections [6-8]. The use of image guidance ensures the injectate is precisely delivered to the ischial bursa thereby enhancing therapeutic efficacy. This was also shown for other interventional pain managements and with different methodologies [9]. In contrast, landmark-based injections, while more convenient, may lead to variable outcomes due to the reliance on anatomical landmarks, which can differ between individuals and be challenging to locate accurately in obese patients or those with altered anatomy.

This study highlights the advantages of fluoroscopic-guided ischial bursa injections over landmark-based approaches for managing refractory ischial bursitis. Patients receiving fluoroscopic injections consistently reported greater pain relief compared to those with landmark-based injections. The variability in pain relief observed with landmark-based injections underscores the importance of patient selection and technique refinement when this method is employed. Overall, this study provides preliminary evidence supporting the use of fluoroscopic-guided ischial bursa injections in patients with refractory ischial bursitis, particularly those who fail to respond to conservative treatments.

This pilot study has several limitations that must be considered. The small sample size of only nine patients restricts the generalizability of the findings, emphasizing the need for larger studies to validate these results. Additionally, the lack of randomization in assigning patients to fluoroscopic-guided or landmark-based injections introduces potential selection bias, which could have influenced the outcomes. The follow-up periods varied and were relatively short, ranging from 4 to 12 weeks, leaving the long-term effectiveness of these interventions unclear. Pain relief was assessed using subjective patient-reported outcomes, which may be influenced by individual expectations or reporting bias. Additionally, this study was conducted at a single institution and included only two treatment groups to evaluate feasibility and methodology, which limits the generalizability of the results.

However, this study provides preliminary evidence supporting the use of fluoroscopic-guided ischial bursa injections in patients with refractory ischial bursitis, particularly those who fail to respond to conservative treatments. Future studies should evaluate longer follow-up periods to validate these findings and explore cost-effectiveness, patient satisfaction, and the potential role of other imaging modalities, such as ultrasound, in guiding injections.

## Conclusions

This small-scale pilot study demonstrates that fluoroscopic-guided ischial bursa injections are associated with significantly higher and more consistent pain relief compared to landmark-based injections in patients with refractory ischial bursitis. The findings suggest that precise medication delivery under imaging may enhance therapeutic outcomes, particularly for patients unresponsive to conservative treatments. Future research should include larger, randomized trials with extended follow-up periods to validate these findings, assess long-term efficacy, and refine treatment protocols for refractory ischial bursitis. Moreover, future studies should be carefully designed and adequately powered to investigate this intervention more rigorously, providing more definitive conclusions.
